# Structure of polymeric nanoparticles encapsulating a drug – pamoic acid ion pair by scanning transmission electron microscopy

**DOI:** 10.1016/j.heliyon.2023.e16959

**Published:** 2023-06-09

**Authors:** Natalia Koniuch, Martha Ilett, Sean M. Collins, Nicole Hondow, Andy Brown, Les Hughes, Helen Blade

**Affiliations:** a School of Chemical and Process Engineering, University of Leeds, Leeds, LS2 9JT, United Kingdom; b School of Chemistry, University of Leeds, Leeds, LS2 9JT, United Kingdom; c AstraZeneca, Oral Product Development, Pharmaceutical Technology & Development, Operations, Macclesfield, SK10 2NA, United Kingdom

**Keywords:** Polymeric nanoparticles, Controlled release through diffusion barrier, PLA-PEG, Cryo-STEM, EELS

## Abstract

Drug-delivery systems based on polymeric nanoparticles are useful for improving drug bioavailability and/or delivery of the active ingredient for example directly to the cancerous tumour. The physical and chemical characterization of a functionalized nanoparticle system is required to measure drug loading and dispersion but also to understand and model the rate and extent of drug release to help predict performance. Many techniques can be used, however, difficulties related to structure determination and identifying the precise location of the drug fraction make mathematical prediction complex and in many published examples the final conclusions are based on assumptions regarding an expected structure. Cryogenic scanning transmission electron microscopy imaging in combination with electron energy loss spectroscopy techniques are used here to address this issue and provide a multi-modal approach to the characterisation of a self-assembled polymeric nanoparticle system based upon a polylactic acid - polyethylene glycol (PLA-PEG) block copolymer containing a hydrophobic ion-pair between pamoic acid and an active pharmaceutical ingredient (API). Results indicate a regular dispersion of spherical nanoparticles of 88 ± 9 nm diameter. The particles are shown to have a multi-layer structure consisting of a 25 nm radius hydrophobic core of PLA and pamoic acid-API material with additional enrichment of the pamoic acid-API material within the inner core (that can be off-centre), surrounded by a 9 nm dense PLA-PEG layer all with a low-density PEG surface coating of around 10 nm thickness. This structure suggests that release of the API can only occur by diffusion through or degradation of the dense, 9 nm thick PLA-PEG layer either of which is a process consistent with the previously reported steady release kinetics of the API and counter ion from these nanoparticle formulations. Establishing accurate measures of product structure enables a link to performance by providing appropriate physical parameters for future mathematical modelling of barriers controlling API release in these nanoparticle formulations.

## Highlights

• Multi-modal cryo-STEM shows 9 nm PLA-PEG diffusion barrier to release of API in polymeric nanoparticle formulation.

## Graphical abstract

**Figure:**
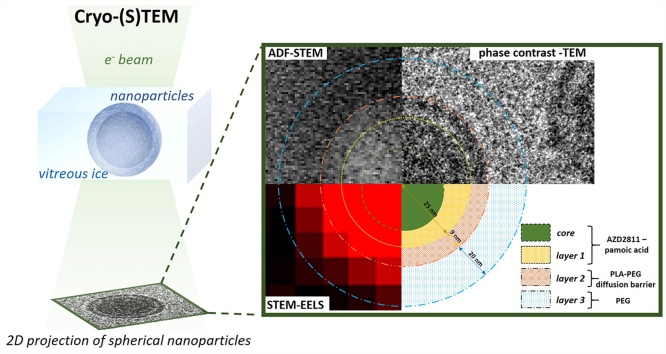

## 1 Introduction

The field of nanomedicine is increasing rapidly as a promising tool to provide target-specific and efficient drug delivery for anticancer therapeutics, but translation into clinical use remains challenging [[1], [2], [3], [4], [5], [6], [7]]. Classical drug delivery via solid formulations such as oral tablets can be limited by poor solubility and low bioavailability of the active pharmaceutical ingredients (APIs). Encapsulation of an API within a functionalized polymeric nanostructure, however, has shown potential as an alternative delivery formulation that could improve therapeutic efficacy by increasing the half-life of drug circulation while reducing side effects [8,9]. One of the promising group of nano-carriers are polymeric core–shell nanoparticles (NPs) because they are able to encapsulate APIs by accumulation in a hydrophobic polymer matrix assumed to be at the core [4,10,11]. A hydrophilic shell provides a hydrated steric barrier to increase the dispersion stability of the NPs in aqueous suspension and to protect them from recognition and removal by the reticuloendothelial system [12], prolonging blood circulation time [13]. The API delivery then depends on the loading efficiency, the rate of diffusion through the polymeric matrix and any degradation of the polymeric NPs themselves [14]. An important aspect of understanding the potential of new drug loaded polymeric NPs is to characterise the particle structure, i.e. identification of the size and morphology of the core-shell nanoparticles and measurement of the distribution of the API within the polymer matrix [15,16].

Polylactic acid - polyethylene glycol (PLA-PEG) polymer NPs have been extensively investigated as anticancer drug delivery vehicles [5,13,17,18]. PLA is commonly used as a drug carrier. However, PLA is hydrophobic and therefore does not disperse well in aqueous suspension, requiring surface modification by hydrophilic PEG to increase the dispersion stability and blood circulation time of the NP form [5]. PLA-PEG NPs are commonly synthesized by the emulsion solvent evaporation or emulsion solvent diffusion methods [13,19]. Multiple studies show high efficiency of encapsulation and conjugation of various APIs (chemotherapy drugs, nucleic acids, peptides) by PLA-PEG based NPs [[19], [20], [21], [22], [23], [24], [25]].

In order to tune nanoparticles to meet a therapeutic profile, analytical techniques provide insight into a physical and chemical structure of the particles and enable the link between nanoparticle structure and product performance to be established [26]. This should ensure that product quality and performance is maintained for the life-time of the drug, yet characterising the complete structure of drug-loaded PLA-PEG polymeric NPs is challenging to do directly at the individual particle level due to a lack of appropriate analytical methods with necessary spatial resolution. Particle size distributions are commonly measured by dynamic light scattering (DLS) and can include surface charge assessment with a zeta potential analyser [19]. The sensitivity of laser scattering of polydispersed particles of significantly different diameters makes interpretation complex and can lead to highly variable results [27,28]. Other analytical techniques such as, optical nanoparticle tracking algorithms, X-Ray scattering or standard electron microscopy (EM) might only measure diameters of a dense particle core, because the hydrated shell is significantly less dense and/or collapsed in high vacuum. The components of PLA-PEG NPs can be distinguished using proton nuclear magnetic resonance (^1^H NMR) by identifying distinct spectral peaks for each synthesized co-polymer [19]. Small-angle neutron scattering (SANS) has shown that the thickness and structure of the PEG layer depends on the molecular weight of the PLA in PLA-PEG NPs [18]. The analysis however involves a model that assumes the core to be homogeneous, have a uniform scattering profile and the shell to be less dense and have a diffuse scattering profile. Pustulka et al. (2013) have used multiple characterisation techniques (^1^H NMR, dynamic light scattering, X-ray Diffraction and cryo-genic TEM) to suggest a three-layer core-shell-corona particle structure to polymeric nanoparticles containing an insoluble low molecular weight compound encapsulated within a particle structure stabilized by amphiphilic diblock copolymers [29]. A polyethylene glycol-*b*-polylactic co-glyocolic acid (PEG-*b*-PLGA) block copolymer was most suitable for potential drug delivery systems, however a PEG corona was assumed to be present rather than being directly identified. Furthermore, the distribution of APIs in these systems is often based on an assumption related to the preparation or polymerization method. For example, Song et al. (2016) reported the potential for use of hydrophobic ion pairing (HIP) of an API (AZD2811) with pamoic acid for controlled release of the API from PLA-PEG NPs, however the structure and distribution of the HIP material in the final NPs was assumed from the preparation method and release kinetics [21,30]. A complete analysis of the structure, chemistry and API loading of these particular PLA-PEG NPs could confirm whether these assumptions are appropriate and provide accurate parameters for predictive modelling of the release kinetics.

**Fig. 1:**
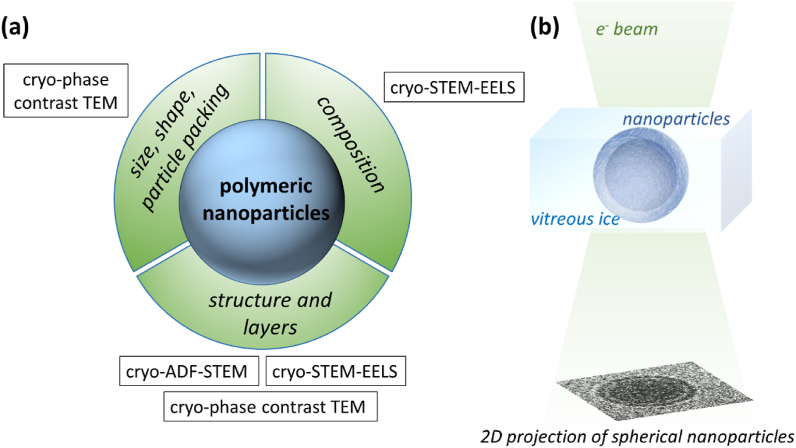
(a) Multi-modal approach to investigate the structure of polymeric nanoparticles using cryo-(S)TEM allowing native state analysis of nanoparticles within a rapidly frozen and vitreous suspension, (b) however acquired cryo-(S)TEM images are always a 2D projection of the spherical nanoparticles.

Scanning transmission electron microscopy ((S)TEM) equipped with an electron energy loss spectrometer can address the challenge of a full characterisation of PLA-PEG NPs because it can determine morphology, structure and chemistry at the single particle level (for a brief electron microscopy background, see Supporting Information 1). Complete native state analysis of NPs is possible by capturing aqueous suspensions in rapidly frozen, vitreous ice followed by transfer into a microscope for cryogenic (S)TEM imaging with electron energy loss spectroscopy (cryo-STEM-EELS) [[31], [32], [33], [34], [35]]. Cryo-phase contrast TEM imaging can be used to analyse the particle size distribution, shape and internal packing [34]. Cryo annular dark field STEM (cryo-ADF-STEM) imaging can complement the phase contrast imaging by identifying changes in material density within particles and potentially composition too [36]. Cryo-STEM-EELS can be used to reveal changes in elemental distribution and chemistry within particles [37]. Thus morphology, size and size distribution, structure and chemical composition of a nanoparticle system can be obtained in one multi-modal analysis at sub particle spatial resolution (Fig. 1a and b). The intrinsic sensitivity of polymers and vitreous ice to high energy electron irradiation does, however, present an additional challenge [35,38,39]. This can be managed by limiting electron fluxes (low-dose conditions) to preserve both the structure and composition of a nanoparticle encapsulated in vitreous ice [32]. To date however, there are no examples of multi-modal STEM characterisation of polymeric nanoparticles at the single particle level despite reports that cryo-TEM can reveal a three-layer core-shell-corona nanoparticle structure of PEG-*b*-PLGA based nanoparticles [40] and, as outlined above, that STEM techniques have the potential to enhance the image contrast of each of these layers.

The present study will investigate the structure and chemistry of a model nanoparticle drug delivery system composed of PLA-PEG NPs where the polymeric matrix encapsulates a hydrophobic ion-pair (salt) between an API (AZD2811) and a counter ion pamoic acid for controlled release of the drug [21]. We use cryo-(S)TEM-EELS for a multi-modal approach to show that these particles form layered structures of different composition and density at the nanometre scale, and we also identify, for the first time, the typical distribution of the API within the particle cores. Our study shows that cryo-(S)TEM, in parallel with standard bulk techniques, can be very useful for checking mechanisms of drug release based on drug diffusion, polymer degradation, and erosion in a nanoparticle system.

## 2 Materials and methods

### 2.1 Sample preparation

Polymeric nanoparticles were obtained from AstraZeneca Ltd. Polymeric nanoparticles containing polylactic acid - polyethylene glycol (PLA-PEG) block copolymer (a number average molecular weight of approximately 16 kDa for PLA and approximately 5 kDa for PEG, determined via nuclear magnetic resonance, NMR), pamoic acid and an active pharmaceutical ingredient API (AZD 2811) were prepared following the method of Song et al. (2016) at an estimated concentration of 137 mg/mL per nanoparticle [21]. The small molecule API contains 19% nitrogen in its molar mass. As produced suspensions potentially contained excess API and residual Tweens (detergents for protein solubilisation), the suspensions were further diluted with ultrapure water (Synergy® purification systems, Type 1, 18.2 MΩ cm at 25 °C) to a final concentration of ∼1.5 mg/mL and stored at 4 °C.

Cryo-TEM specimens were obtained by placing 3.5 μL of the diluted polymeric nanoparticle solution onto plasma cleaned lacey carbon TEM grids. Each grid was blotted and rapidly plunge frozen into liquefied ethane using a FEI Vitrobot© and transferred into a Gatan 914 TEM cryo-holder under liquid nitrogen. The temperature was kept to be below −170 °C during transfer and subsequent TEM observation.

### 2.2 Cryogenic transmission electron microscopy

Cryo-(S)TEM-EELS analysis was performed using a ThermoFisher Titan^3^ Themis G2 operated at 300 kV, equipped with a monochromator, Gatan Quantum 965 ER spectrometer and a Gatan OneView camera. Bright-field (BF-TEM) images and selected area electron diffraction (SAED) patterns were acquired in a low-dose condition not exceeding a total of ∼10–20 e^−^/Å^2^ per acquired image. Initial bright field TEM (BF-TEM) observation was carried out using 9.8 *kx* magnification with 0.85 nm pixel size and 4096 by 4096 pixel image size and the electron fluence was set to be ∼0.8 e^−^/Å^2^s giving a cumulative electron fluence of ∼2 e^−^/Å^2^ per image. Annular dark field (ADF-STEM) was undertaken using a probe current of 40 pA and a 40 μs dwell time and the magnification limited to 57 *kx* giving a total accumulated fluence of ∼60 e^−^/Å^2^ per pixel for each acquired image. Detailed experimental conditions for each presented image in the figures throughout this manuscript are summarised in Supporting Information 2.

Cryo-STEM-EELS spectrum imaging was undertaken using the Gatan Quantum 965 ER imaging filter in the image-coupled mode. To minimize specimen damage, vitreous ice melting, and subsequent drift, moderate dose was used to collect spectrum imaging data at an acquisition fluence of 715 e^−^/Å^2^ per pixel (see details in Supporting Information 2).

## 3 Results

### 3.1 Imaging condition optimisation

**Fig. 2:**
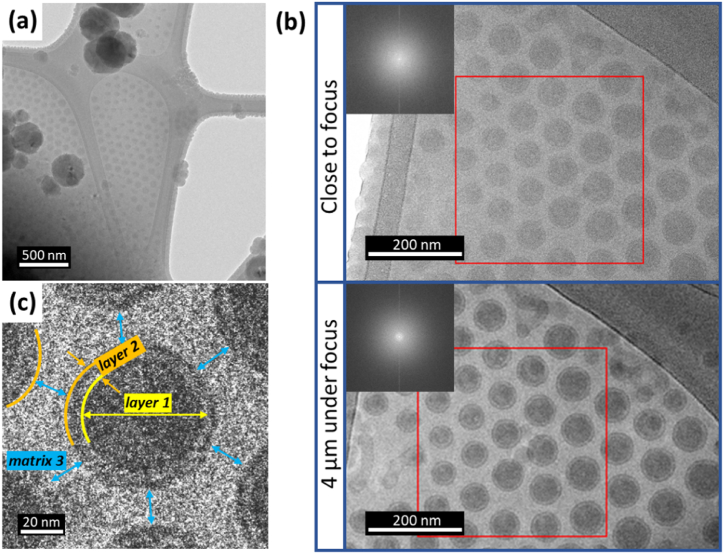
Cryo-TEM micrograph of polymeric nanoparticles: (a) Low magnification BF-TEM micrograph taken close to focus and (b) close to focus high magnification image (top right micrograph) and the effect of applying under-focus to the same area (bottom right micrograph) in order to increase the particle contrast to allow (c) identification of three individual layers in each nanoparticle (as labelled in the image).

There is no observation of particle overlap in the Z-direction in any of the acquired images (Fig. 2a). The similarly sized polymeric nanoparticles self-organise into reasonably regular hexagonal close-packed 2D arrays and it is clear that most are separated by a constant spacing, suggesting the presence of either significant surface charge on the nanoparticles or a low electron density layer of material between the particles rather than just ice. As seen in Fig. 2a, the polymeric nanoparticles have low contrast in the in-focus BF-TEM image due to their density being similar to that of the vitrified ice background.

The magnification and contrast of these images is not enough to enable characterisation of the molecular packing/structure of the polymeric NPs. In order to begin to characterise the structure of the nanoparticles, the magnification was increased (Fig. 2b). The electron fluence for this magnification was set to be ∼9.7 e^−^/Å^2^s giving the cumulative electron fluence of ∼10 e^−^/Å^2^ per image. In Fig. 2b, the same area was imaged close to Gaussian focus and with 4 μm under focus (bottom) to enhance particle contrast (PC) of the TEM image [29]. In the image acquired close to focus, the particles have low contrast and a boundary layer between a core and a surface layer is barely visible. At the under-focus condition, a lower density surface layer surrounding the particle core becomes more visible (layer 2 in Fig. 2c). De-focusing increases the image contrast but potentially might affect the measurable size of the NPs due to the generation of an under-focus Fresnel fringe. The differences between the mean values of the in- and under-focus particle diameters (outer, low density layer) were shown to not differ by more than 0.7 nm. However, determination of the boundaries of NPs in the image close to focus is difficult. An estimate of a manual measuring error of ±2 nm in diameter size (under/over-estimate of the size of the NPs) was used and in-focus values compared to the equivalent under-focus image values (Supporting Information 3). A two-tailed *P*-test run on these measured diameters yielded P = 0.097, suggesting that the difference in the measured mean value between the image types is not statistically significant (P > 0.05) and that diameters measured at 4 μm under-focus can be used for further analysis. Thus, three components to each polymer nanoparticle are easily identified in BF-TEM images: (i) layer 1 – a core, (ii) layer 2 – a coating of the core and (iii) an assumed matrix (layer) 3 – which must be a low density coating or charge-limited space around each particle that is preventing particle aggregation (Fig. 2c).

### 3.2 Critical fluence measurement

**Fig. 3:**
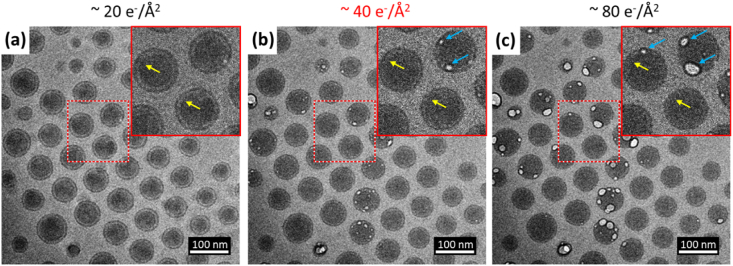
Electron beam damage observation in cryo-BF-TEM based on imaging the same area with a total electron fluence of (a) 20 e^−^/Å^2^, (b) 40 e^−^/Å^2^ and (c) 80 e^−^/Å^2^. Yellow arrows indicate the layer 1 boundary which begins to fade at 40 e^−^/Å^2^ indicating damage to the structure of the nanoparticles. The blue arrows indicate bubble formation, especially at the interface between layer 1 and layer 2, related to severe radiation damage. Based on the observation of the damage propagation, <40 e^−^/Å^2^ (marked in red font) is considered as a safe dose for cryo-TEM imaging. (For interpretation of the references to colour in this figure legend, the reader is referred to the Web version of this article.)

Low dose selected area electron diffraction and high magnification images do not reveal any signs of crystallinity in the polymeric NPs (data not shown). To asses sample integrity, an electron beam damage threshold was estimated by identifying changes to particle structures in a series of images collected at progressively higher cumulative electron fluence of (a) 20 e^−^/Å^2^, (b) 40 e^−^/Å^2^ and (c) 80 e^−^/Å^2^ (Fig. 3a–c). Electron beam damage of the particles is identifiable by two characteristic features. First, a decrease in contrast between layer 1 and 2. Yellow arrows in the higher magnification insets of Fig. 3 indicate that the layer 1 boundary begins to fade at 40 e^−^/Å^2^. Second, void formation preferentially occurred at the interface between layer 1 and layer 2 (marked by blue arrows in the higher magnification inset). Voids or bubbling in polymeric material can result from restricted diffusion of radiolysis products i.e. formation of gas (e.g. hydrogen H^+^) within a vitrified sample [41]. As cumulative electron dose increases, the bubbling expands in layer 2 and some bubbling can also be observed in layer 1 (although this could be from layer 2 on the top surface of the projected image of the spherical nanoparticles, Fig. 1b). This suggests a structural difference between layer 1 and 2 consistent with the image density difference. Layer 2 is made of the most beam sensitive component and damage products may migrate to the interface between layer 1 and 2 where the interface acts as a nucleation point for gas bubble formation. It is not possible to get a robust critical fluence value from the limited image series, however based on the damage observation, the cumulative electron fluence per image in cryo-TEM should be kept below 40 e^−^/Å^2^.

### 3.3 Physical characterisation

**Fig. 4:**
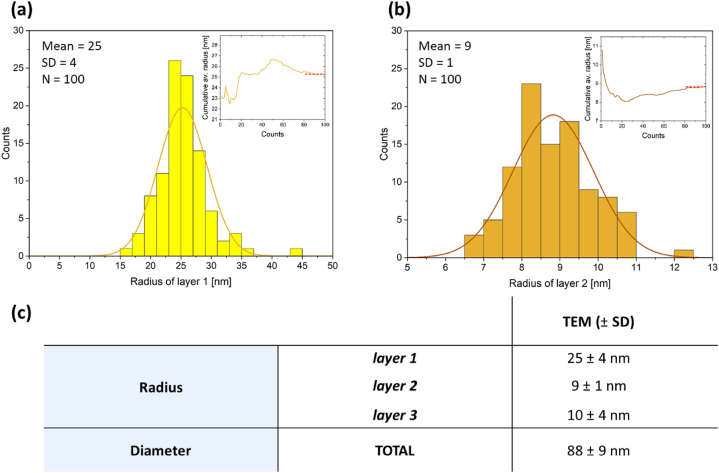
(a)–(b) Particle size distribution histograms of radius of layer 1 and thickness of layer 2 measured on the defocused images, respectively. The inserts represent the cumulative mean radius, suggesting that a minimum of 80 particles is required to accurately measure the particle size distribution (since after that number the cumulative radius does not vary significantly). (c) Table with the mean values for layers 1–3. The average diameter of nanoparticles is 88 ± 9 (±SD) nm.

**Fig. 5:**
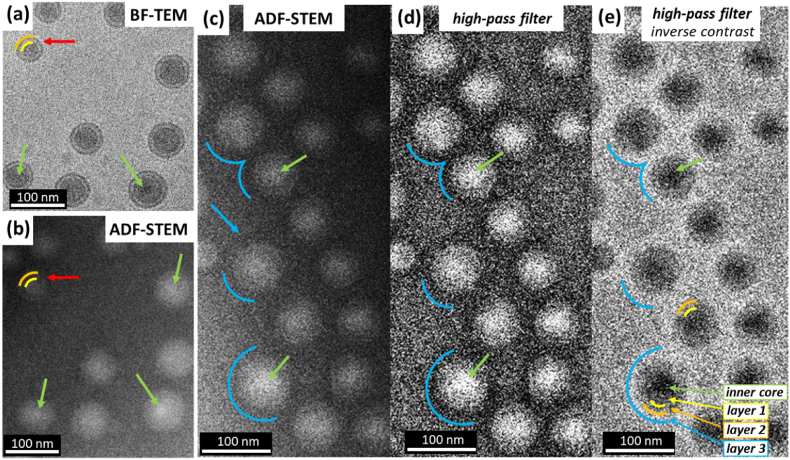
Cryo-ADF-STEM micrograph of polymeric nanoparticles showing bright central regions in the core of some nanoparticles (green arrows) that might suggest a high density region within an inner core of the nanoparticles. (a)–(b) comparison between under-focus BF-TEM and ADF-STEM of the same region. Yellow curves indicate layer 1 and orange curves indicate layer 2. (c) A dark halo is visible around the particles in the ADF-STEM image (blue arrow and curves) suggesting the presence of layer 3 and it being lower density than the core and the surrounding vitreous ice. d) Application of a high pass filter to the ADF-STEM image removes thickness variation and emphasizes the low electron density of layer 3 and e) inverting the contrast of the high pass filtered ADF-STEM image reveals all three layers of the core-shell-corona nanoparticles. (For interpretation of the references to colour in this figure legend, the reader is referred to the Web version of this article.)

As discussed above, the under-focus BF-TEM images may be used for representative size distribution measurement, of both the entire nanoparticle size and the thickness of the individual layers. The particle size distribution, determined from 100 manually measured nanoparticles in ImageJ [42], is presented in Fig. 4a–c. Individual layers were measured by the following procedure (shown schematically in Fig. S3, Supporting Information 4). (i) Layer 1 - the radius is half the maximum Feret diameter. (ii) Layer 2 – the thickness is half of the difference between the two Feret diameters of layer 1 and 2. (iii) Layer 3 - the thickness is taken to be half of the smallest distance between adjacent particles. We acknowledge that layer 3 appears as a matrix with no change in electron density between this and the surrounding ice by cryo-TEM (Fig. 2). We know however that there must be charge separation or some organic material in this region that is preventing the particles from aggregating and our subsequent analysis will confirm this to be a distinct carbon containing layer (Fig. 5, Fig. 7, Fig. 8). The mean radius for layer 1 was 25 ± 4 (±SD) nm, and the thickness for layer 2 was 9 ± 1 (±SD) nm and for the majority of particles, the thickness of layer 3 was 10 ± 4 (±SD) nm. The total mean diameter of the polymeric nanoparticles was 88 ± 9 (±SD) nm (layer 1–3), over a size range of 68–126 nm. The insets (Fig. 4a and b) show cumulative mean radius against particle counts, indicating that a consistent particle size was obtained after 80 or more particle measurements.

Annular dark-field STEM (ADF-STEM) imaging was performed to further investigate the structure of the nanoparticles, particularly to reveal the presence of any material other than vitreous ice in layer 3 and to see if the API was detectable in layer 1 or 2. As the nanoparticles consist only of light elements, i.e. hydrogen, carbon and oxygen, the additional scattering contribution from nitrogen that is present in the API, might generate extra contrast in an ADF image of the core. Fig. 5 shows cryo-ADF-STEM images of the polymeric nanoparticles and these do indicate bright regions (higher contrast) towards the centre of layer 1 of some particles, marked by green arrows and suggesting the presence of an inner core. In corresponding cryo-phase contrast-TEM and cryo-ADF-STEM micrographs, layer 1 and layer 2 boundaries can be seen and are marked by yellow and orange curves, respectively (Fig. 5a and b). Moreover, a dark (lower contrast) halo is visible around many particles suggesting layer 3 contains material of lower scattering density than both the particle cores (layers 1 and 2) and the surrounding vitreous ice (the upper boundary of layer 3 is marked by a blue arrow and curves in Fig. 5c). The thickness of these ‘halo’ regions is measured to be ∼10 nm and this is in good agreement with the previously measured layer 3 thickness from cryo-phase contrast-TEM images. To reduce the impact of thickness variation of the vitreous ice in the ADF-STEM images a high pass filter is applied (Fig. 5d). This confirms a lower electron density for layer 3 than the surrounding ice and inversing the contrast clearly reveals the inner core, layer 1, 2 and 3 in one image i.e., direct identification of a core, shell and corona particle structure (Fig. 5e). A particle corona of lower electron density than vitreous ice could be due to there being a low density brush of PEG molecules attached to layer 2 only. A 5 kDa PEG chain typically extends out by 10 nm for a dense brush layer [43] however this may extend further to 10–20 nm when the brush packing becomes less dense [44,45].

### 3.4 Low-dose EELS for chemical characterisation

**Fig. 6:**
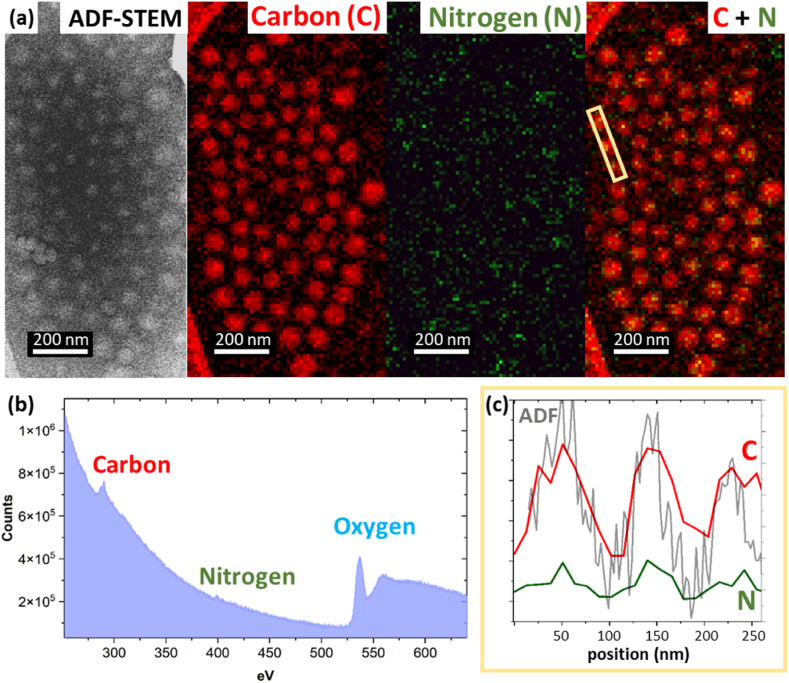
(a) Cryo-ADF STEM and cryo-STEM EELS carbon and nitrogen mapping. (b) Cryo-STEM-EEL spectrum acquired over the whole area shows the presence of C, N and O. (c) A linear intensity profile taken from the C–N overlay map and ADF-STEM image indicates that C occurs also between the nanoparticles and N is located approximately in the centre of individual nanoparticles (yellow box in (a)). (For interpretation of the references to colour in this figure legend, the reader is referred to the Web version of this article.)

As outlined above, cryo-BF-TEM and cryo-ADF-STEM were used to identify the three layers plus an inner core to the polymeric NPs. However, elemental spectroscopy is needed to complete chemical identification and to locate the nitrogen-containing API. In order to maintain a low dose condition, cryo-STEM-EELS elemental maps were acquired with a 12 nm spatial resolution sufficient to identify the thinnest component of the structure identified so far i.e. layer 2. Fig. 6a–c presents the STEM-EEL spectra that reveal the characteristic C–*K*, N–*K* and O–*K* edges from within the particles, and from which the elemental maps for carbon and nitrogen were gathered. The spatial distribution of the C signal is in good agreement with the scattering contrast from these carbon rich, polymeric NPs seen in the corresponding cryo-ADF-STEM image. Line profiles along C and N elemental overlay maps confirm that the peak C and N signals within nanoparticles correspond to the bright central region or inner core identified in the cryo-ADF-STEM images, suggesting, that these are where the N-rich API is located.

**Fig. 7:**
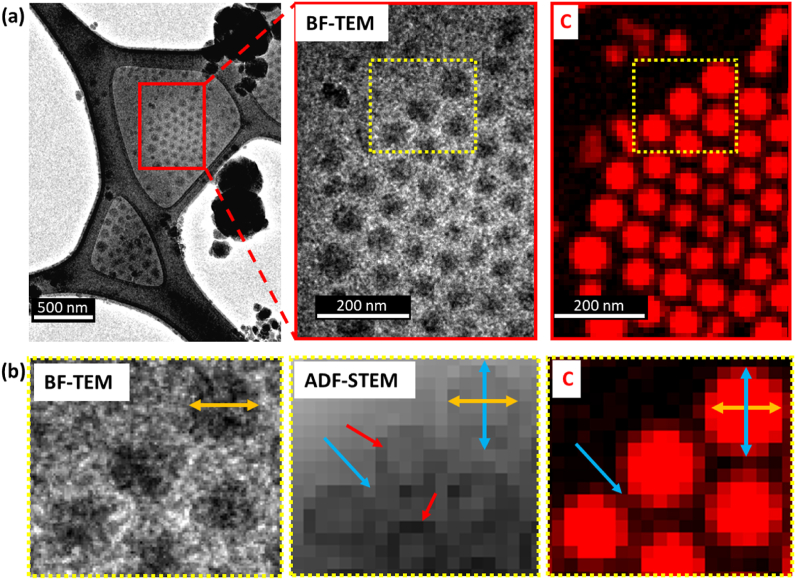
(a) Under-focus BF-TEM image and PCA treated carbon STEM-EELS mapping of the area marked in red. (b) Under-focus BF-TEM, ADF-STEM and C-*K* EELS mapping of a cropped fragment marked in yellow in (a) and showing carbon presence between the particles, confirming the C-content to layer 3. The corresponding diameter of layer 1 + 2 is identical between cropped under-focus BF-TEM, ADF-STEM and C-*K* map images (yellow arrows) and the thickness of layer 3 is consistent with earlier measurements of the spacing between nanoparticles by under-focus BF-TEM (blue arrow). Red arrows indicate severe electron beam damage in layer 2 visible in a post-EELS acquisition, ADF-STEM image. (For interpretation of the references to colour in this figure legend, the reader is referred to the Web version of this article.)

**Fig. 8:**
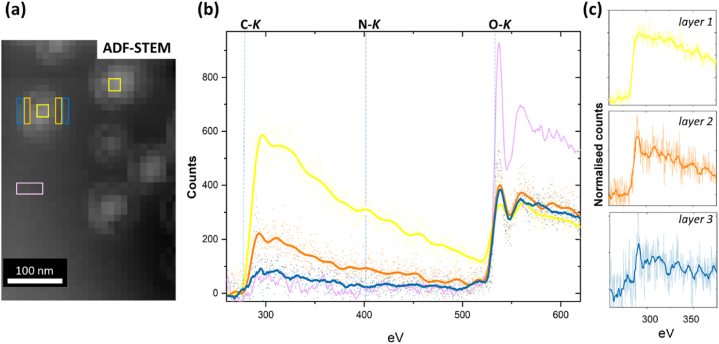
(a) Cryo-ADF-STEM image of particles and areas where spectra were extracted from – layer 1 (yellow box), layer 2 (orange boxes), layer 3 (blue boxes) and amorphous ice (purple box). (b) Background stripped, as-acquired (faint dots) and smoothed (bold lines) STEM-EELS from layer 1, layer 2 layer 3 and amorphous ice areas in (a). Layer 1 is C rich and also has a clear N peak identifiable. Layer 2 and 3 have progressively less C and relatively more O per layer and less than that of the amorphous ice. (c) Background stripped, as-acquired (faint lines) and smoothed (bold lines) STEM-EELS at the C-*K* edge reveal that layer 2 and layer 3 have slightly different edge structure to that of layer 1 suggesting a different polymer composition. (For interpretation of the references to colour in this figure legend, the reader is referred to the Web version of this article.)

Low-dose cryo-STEM-EELS elemental maps can be de-noised by application of Principal Component Analysis (PCA). Particular attention was made to the correct identification of significant components in the corresponding scree plots (Supporting Information 5). An example of a PCA de-noised STEM-EELS carbon elemental map is shown in Fig. 7a. The corresponding under-focus BF-TEM, ADF-STEM and C elemental maps allow for multi modal interpretation and identification of the structural components of the nanoparticles. The diameter of layer 1 plus layer 2 measured by under-focus BF-TEM image is in good agreement with the diameter measured by ADF-STEM and C mapping (orange arrow). However, layer 3, which is not directly seen by under-focus BF-TEM, can be identified by ADF-STEM (Fig. 5) and C mapping (blue arrows in Fig. 7b) and is consistent with a 10 nm thickness layer containing organic material, as inferred from the spacing of the NPs in BF-TEM (Fig. 2). Moreover, layer 2 was significantly damaged during EELS mapping and is seen as a dark rim to the NPs in the post-acquisition ADF-STEM image (red arrows in Fig. 7b) confirming the higher electron beam sensitivity of this component compared to layer 1 i.e., that layer 2 is of different composition to layer 1. Layer 3 does contain C but it is at a significantly lower concentration than in layers 1 and 2 and damages at the same rate as the surrounding ice.

Averaged STEM-EEL spectra were selected from particle layers 1, 2 and 3 and are presented in Fig. 8. All areas were of equal size. The smoothed spectra show the C–*K* and O–*K* edges are visible in all layers and the N–*K* edge is clear detectable in layer 1 (Fig. 8a and b; note that trace C may be detected in the amorphous ice but at levels lower than that in layer 3). The maximum C counts in unsmoothed spectra of the measured areas for the spatially separated layers decreases from 600 for layer 1 to 240 for layer 2 and 120 for layer 3. The C:O peak intensity ratio varies from 1 to 0.7 for layer 1, to 1 to 2.1 for layer 2 and 1 to 4.1 for layer 3 (and the absolute O count in layer 3 remains lower than that in the amorphous ice plus the ice has a C:O ratio of 1–11.2). These data are consistent with the TEM and ADF-STEM results that show the core or layer 1 of the nanoparticles has the most dense C packing and contains the majority of the API, and that the very outer layer (3) is the least dense but does indeed contain C. The near edge fine structure (or shape) of the un-smoothed, background subtracted C-*K* edges are shown in Fig. 8c. Layer 1 C-*K* edge shows a broad C 1s−π* peak just above 285 eV suggesting additional transitions to the C 1s−π*C═C peak. These may result from unsaturated bonds introduced by electron beam damage during irradiation [33]. This would be consistent with the C-rich, conjugated C structures of pamoic acid and the API being in layer 1, as also revealed by the N mapping (Fig. 6). The C 1s−π* peak is much sharper in layer 2 suggesting a simpler carbon structure than layer 1 consistent with layer 2 being dominated by the more simple PLA and/or PEG structures. The C *K*-edge for layer 3 is noisy but arguably closest in shape to that of layer 2.

**Fig. 9:**
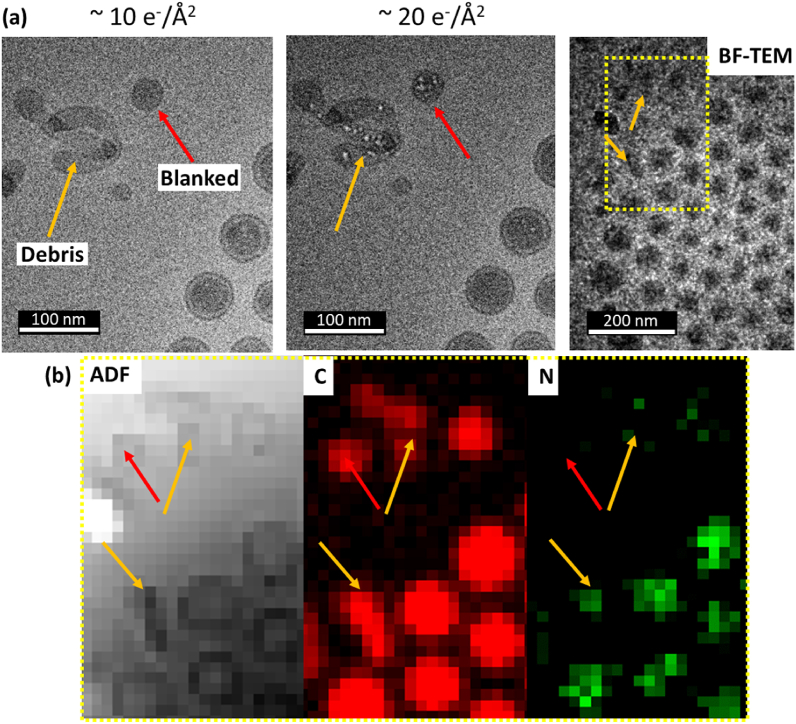
(a) Under-focus BF-TEM of non-standard nanoparticles named as: debris (non-spherical particles, marked as orange arrows) and blank (no visible layer 1, marked as red arrows). Both non-standard particles are highly beam sensitive. (b) Cropped cryo-STEM-EELS mapping of area marked in yellow in (a) suggests that debris and blank particles consist of the same material as layer 2 in the standard particle (i.e., have negligible N content and are highly beam sensitive). (For interpretation of the references to colour in this figure legend, the reader is referred to the Web version of this article.)

In addition to the major structural layering of the polymeric nanoparticles, two types of distinct, non-standard particles have been identified: (i) ‘Blank’ - circular particles with no visible layer 2, and (ii) ‘Debris’ - non-circular particles of various shapes (Fig. 9a). Based on electron beam damage observations of these, it can be seen that in both types, the same damage mechanism operates to that in the case of layer 2 i.e. void or bubble formation throughout these particles. This suggests that these non-standard particles are made of the same, very beam sensitive material as layer 2 which is inferred to not contain significant amounts of the N-rich API. STEM-EELS C and N elemental mapping show that the 'Debris' and ‘Blank’ particles do indeed contain only a negligible amount of nitrogen (and so have little or no API content) (Fig. 9b).

## 4 Discussion

**Fig. 10:**
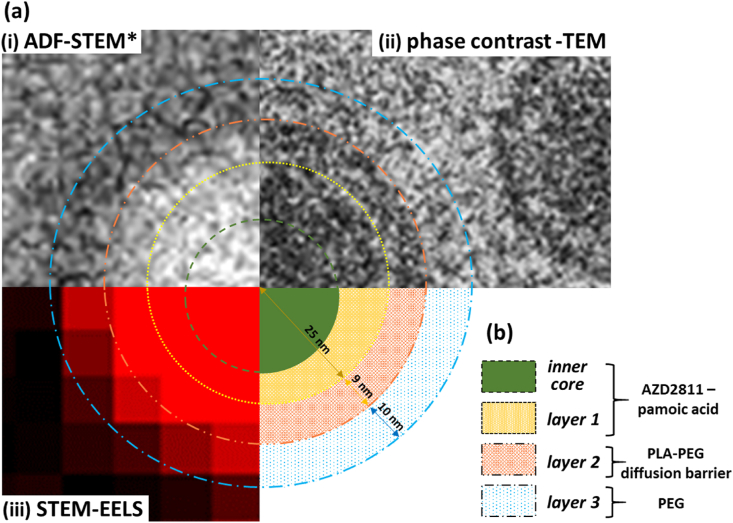
(a) Final multi-modal data-driven model of the polymeric nanoparticles based on (i) ADF-STEM (*high-pass filtered), (ii) phase contrast-TEM and (iii) STEM-EELS analysis. (b) The likely content of each layer is -pamoic acid-API and some PLA material in layer 1 with a core enriched in pamoic acid-API material. Only PEG and PLA in layer 2 and diffuse PEG packing in layer 3.

Combining the results of the cryo phase contrast-TEM, ADF-STEM and STEM-EELS, we put forward a final model of these polylactic acid (PLA), polyethylene glycol (PEG), pamoic acid and active pharmaceutical ingredient (API) polymeric NPs (Fig. 10a and b). Starting from the centre of the polymeric NP, the following structural components have been identified: inner core within layer 1, layer 1, layer 2 and layer 3. We also know that the nano-emulsion synthesis method will result in self-assembly of the components such that the hydrophobic material must end up coated by the hydrophilic PEG when a stable colloid is achieved as is the case here. Taking only this information we can begin to piece together the identity of the layers.

ADF-STEM shows a bright, sometimes off-centre inner core that was confirmed by STEM-EELS elemental maps to contain N (Fig. 6) and so can be related to the highest concentration of the API in the nanoparticle. The inner core occupies only a part of layer 1 that was itself identified by BF-TEM to have a radius of 25 nm ± 4 (±SD) nm (Fig. 2, Fig. 4). This layer does not show electron beam damage or bubbling at or just above the critical fluence of 40 e^−^/Å^2^ (Fig. 3). It is the most C-rich and dense of all the layers by ADF-STEM and EELS mapping (Fig. 5, Fig. 6, Fig. 7), it has a broad C 1s−π* peak by EELS (Fig. 8), indicative of a range of carbon bonding in the core. This is consistent with the presence of the C-rich pamoic acid and API throughout layer 1 because both contain conjugated carbons that are more resistant to electron beam damage than linearly bonded polymer chains [46]. We also know however that the self-assembly will occur around a hydrophobic polymer blend and so we can expect that the PLA part of the PLA-PEG co-polymer will be attracted to the hydrophobic ion-paired pamoic acid and API in layer 1.

A layer 2 of different contrast to layer 1 is identified around the NPs by under-focus BF-TEM and its thickness is 9 ± 1 (±SD) nm (Fig. 2, Fig. 4). Layer 2 is made of a more beam sensitive component than layer 1 (Fig. 3) and is of slightly lower density than layer 1 (because of lower contrast in the de-focussed BF-TEM images that is also seen by inverted contrast, high-pass filtered ADF-STEM; Fig. 5d and e). STEM-EELS confirms this layer is less C-rich and has simpler C structure than layer 1 (Fig. 8). Thus, we could conclude this layer contains little or no pamoic acid-API pairing i.e. no HIP material. This is consistent with the increased beam sensitivity of this layer (Fig. 3; [34]). Thus, layer 2 could consist of a layer of PLA or a layer of mixed PLA-PEG assembly or even a layer of PEG. Both PEG and PLA polymers damage by bond scission and release of free radicals that could lead to gas formation and bubbling as seen here [33]. It is not clear if the PLA-PEG co-polymer remains intact during self-assembly, but if it does then one would expect the hydrophobic PLA to phase separate or align to the HIP material during synthesis to form a distinct layer. We might then suggest layer 2 could be richer in PLA or alternatively layer 2 is a mixed PLA-PEG layer HIP core on self-assembly. Ultimately PEG is the hydrophilic component and will assemble at the surface of the NPs. A PEG layer however is generally shown to be difficult to directly visualise by TEM due to low density packing of the PEG at the surface [29,47].

The final layer 3 was identified by all methods to have a nominal thickness of 10 nm ± 4 (±SD) nm however it was not directly visible by BF-TEM (Fig. 2) but was identified in high-pass filtered ADF-STEM images and was shown to be of lower electron density than both the NP cores and vitreous ice (Fig. 5c, d and e). Layer 3 was shown by STEM-EELS to contain some C and of similar bonding structure to layer 2 (Fig. 7, Fig. 8). We suggest therefore that this layer contains a loosely packed, brush of hydrophilic PEG chains that provide the main stearic dispersion of the NPs at a regular, minimum physical distance of 20 nm (Fig. 2). The 5 kDa PEG chains would be anchored to layer 2 and so could extend out 10–20 nm depending on their packing density [[43], [44], [45]].

We can now link these structural findings back to prior reports on the performance of these nanoparticle formulations. For example, Song et al. (2016) suggest that the key factor in loading and API release is the counter ion pairing and we confirm here the co-localization of the two within the 50 nm diameter core of these particles (layer 1) [21]. They also suggest steady-state co-diffusion and co-release of the pamoic acid and API without an initial burst release of surface adsorbed material from these formulations. Our results provide mechanistic understanding of these release kinetics – we have shown that the majority of the pamoic acid and API is located within an inner core in layer 1 and so release will require diffusion through or degradation of the outer edges of the core and through the dense, 9-nm-thick layer 2, which is rich in PLA-PEG. Thus, knowing the size of the API-rich inner core plus the thickness of layer 2 and the molecular weight of the PLA-PEG copolymer gives starting parameters for future mathematical modelling of a diffusion barrier inhibiting API release [26]. Making the link between nanoparticle structure and product performance enables the structure of the nanoparticles to be optimised and for robust control strategies to be established to ensure that product quality and performance is maintained for the life-time of the drug.

## 5 Conclusions

A multi-modal cryo-(S)TEM approach provides physical and chemical characterisation that can be linked to the release kinetics of a model polylactic acid - polyethylene glycol (PLA-PEG), and hydrophobic ion-pair between pamoic acid and an active pharmaceutical ingredient (API) polymeric nanoparticle system. Low-dose cryo-(S)TEM, without staining or chemical fixation, is shown to provide characterisation in the native state. Phase contrast-TEM in combination with STEM imaging and elemental analysis reveals a three-layer structure to these self-assembled particles consisting of a 25 nm radius hydrophobic core of PLA and pamoic acid-API material with additional enrichment of the pamoic acid-API material within the inner core (that can be off-centre), surrounded by a 9 nm thick, dense PLA-PEG layer all with a low density PEG surface coating of around 10 nm thickness. This structure suggests that release of the API can only occur by diffusion through, degradation of or both processes simultaneously acting on the 9 nm thick, dense PLA-PEG layer, with diffusion being the process most consistent with the previously reported steady release kinetics of API and counter ion from these nanoparticle formulations. Establishing this link between product structure and performance provides appropriate physical parameters for future mathematical modelling of the PLA-PEG diffusion barrier controlling API release in these nanoparticle formulations.

## Author contribution statement

**Natalia Koniuch, Nicole Hondow, Andy Brown, Les Hughes and Helen Blade**: Conceptualization, **Natalia Koniuch, Martha Ilett, Sean Collins, Nicole Hondow and Andy Brown:** Methodology, Validation, Formal Analysis, Investigation, **Les Hughes and Helen Blade**: Resources, **Natalia Koniuch**: Data curation, **Natalia Koniuch and Andy Brown**; Writing- Original draft preparation, **All authors**: Writing- Reviewing and Editing.

## Data availability statement

The data that support the findings of this study are available from the corresponding author upon reasonable request.

## Declaration of competing interest

The authors declare the following financial interests/personal relationships which may be considered as potential competing interests:Natalia Koniuch reports financial support was provided by 10.13039/501100000266Engineering and Physical Sciences Research Council Grant no 2182593.

## Abbreviations

API: active pharmaceutical ingredient
cryo-ADF-STEM: cryogenic annular dark-field in the scanning transmission electron microscope
cryo-BF-TEM: cryogenic bright-field transmission electron microscopy
cryo-STEM: cryogenic scanning transmission electron microscopy
cryo-STEM-EELS: cryogenic scanning transmission electron microscopy coupled with electron energy loss spectroscopy
cryo-TEM: cryogenic transmission electron microscopy
EELS: electron energy loss spectroscopy
HIP: hydrophobic ion pairing
NP: nanoparticle
NPs: nanoparticles
PCA: principal component analysis
PEG: polyethylene glycol
PLA-PEG: polylactic acid - polyethylene glycol
PLA: polylactic acid
SAED: selected area electron diffraction
STEM: scanning transmission electron microscopy
TEM: transmission electron microscopy
