# Supplementary material for: Structure of polymeric nanoparticles encapsulating a drug – pamoic acid ion pair by scanning transmission electron microscopy
Source: Heliyon. 2023 Jun 9;9(6):e16959. doi: 10.1016/j.heliyon.2023.e16959 (PMC10285183; doi:10.1016/j.heliyon.2023.e16959)
Supplement: Multimedia component 1 [file mmc1.docx]

**Supplementary Information for**

**Structure of Polymeric Nanoparticles Encapsulating a Drug – Pamoic Acid Ion Pair by Scanning Transmission Electron Microscopy**

Natalia Koniuch^†^, Martha Ilett^†^, Sean M. Collins^†,‡^, Nicole Hondow^†^ and Andy Brown ^†^

Les Hughes^§^ and Helen Blade ^§^

^†^ School of Chemical and Process Engineering, University of Leeds, Leeds LS2 9JT, United Kingdom

^‡^ School of Chemistry, University of Leeds, Leeds LS2 9JT, United Kingdom

^§^ AstraZeneca, Oral Product Development, Pharmaceutical Technology & Development, Operations, Macclesfield SK10 2NA, United Kingdom

**Supporting information 1**

**Electron microscopy background**

In a transmission electron microscope (TEM), the electron beam is transmitted through a thin specimen (electron transparent) to produce 2-D images of the projected shape and internal structure of 3-D objects [1, 2]. A high energy beam of electrons is used instead of light to achieve higher spatial resolution, requiring the maintenance of high vacuum in the column of the microscope to prevent electron collision with gas molecules. In TEM, electrons are emitted by an electron gun, focused into a small, coherent and parallel beam by a series of condenser lenses and this beam is then transmitted through the specimen where a number of interactions can occur (Figure S1a). The scattered electrons can be collected, focused and magnified by a set of lenses in order to produce an image of the projected shape and internal structure of a specimen. TEMs have an intermediate diffraction lens, thus an electron diffraction pattern can be acquired in order to study the crystal structure. Moreover, other interaction products e.g. X-rays can be collected by a corresponding detector or spectrometer that enables elemental analysis of a specimen. The contrast formation in TEM images is generally produced by a combination of specimen mass and thickness variation, crystalline or diffraction scattering and phase differences in the transmitted electrons. Moreover, a TEM can operate at different magnifications, thus allowing quick characterisation of nano, micro and macro scale features of a material.

Another approach to achieve similar image resolution is by scanning transmission electron microscopy (STEM) [1, 2]. Many conventional TEMs can operate in STEM imaging mode, wherein instead of parallel illumination used by TEM, a converged beam is focussed to a nanoscale or Ångstrom-scale electron probe, and an image is generated by use of scanning coils to raster the probe across the specimen (Figure S1b). The scattered electrons can be collected at each probe dwell point by post-specimen detectors: (i) bright-field (BF) detector (collecting electrons scattered at <10 mrad and can be used for phase contrast imaging which by reciprocity can be similar to a conventional TEM image), (ii) annular dark-field (ADF) detector (collecting electrons scattered to higher angle than BF but lower angle that HAADF and can be used for imaging materials with different density and possible imaging lighter elements); and (iii) high-angle annular dark-field (HAADF) detector (collecting high angle, >50 mrad, incoherently scattered Rutherford electrons and used for Z-contrast imaging).

Figure S1 shows a comparison of beam propagation in TEM and STEM modes.

STEM can be coupled with electron energy loss spectroscopy (EELS) (Figure S1b). Therefore, it is possible to collect high spatial spectroscopy information with HAADF/ADF signal simultaneously [3, 4]. As the high energy electron beam is transmitted through thin specimen, some electrons can interact with the sample via energy exchange (i.e. inelastically). This energy lost by the incident electrons can be measured by an EELS spectrometer and detector sitting below the BF detector (Figure S1b). The acquired electron energy loss spectrum can provide structural and chemical information about the sample e.g. band structure and specimen thickness (zero-loss and plasmon peak), elemental composition (core-loss edges) or bonding and oxidation states (electron energy loss near edge fine structure) (Figure S1c). It is also possible to acquire spectral information at each probe (pixel) position (X,Y), and thus a spectrum image (STEM SI) can be collected and used for elemental mapping.

As (S)TEM requires a high vacuum in the microscope column, analysis of hydrated materials requires freezing of a thin film of specimen to prevent dehydration, ideally in a vitrified (amorphous) form to prevent freezing (crystallization) induced redistribution of solute. Recent development in cryogenic electron microscopy (cryo-EM) allows investigation of soft matter and biological samples at the near native state, and the single particle reconstruction technique has been awarded a Nobel Prize in 2017 [5]. In cryo-EM, it is important to prepare cryo-EM grids with a thin, well dispersed distribution of particles of interest within an aqueous suspension by a rapid vitrification process (to prevent ice formation), to provide structural and chemical information of hydrated samples [6]. Schematic representation of cryo-EM sample preparation is presented in Figure S2. Briefly, a drop of suspension (3 – 5 μL) is loaded onto plasma treated, thin carbon film EM grid (plasma treated to make the surface of the film hydrophilic) and blotted to form a thin layer of the aqueous suspension and sample (Figure S2a, ‘A’). Then, the EM grid is rapidly plunge frozen into liquefied ethane (Figure S2a, ‘B’), transferred into a cryo-transfer holder under liquid nitrogen (Figure S2b) and loaded, cold into the microscope. This vitrified thin film containing a dispersion of the particles of interest can be then analysed via cryo-(S)TEM techniques, preferentially of a suspension over a hole in carbon film in the EM grid (Figure S2c).


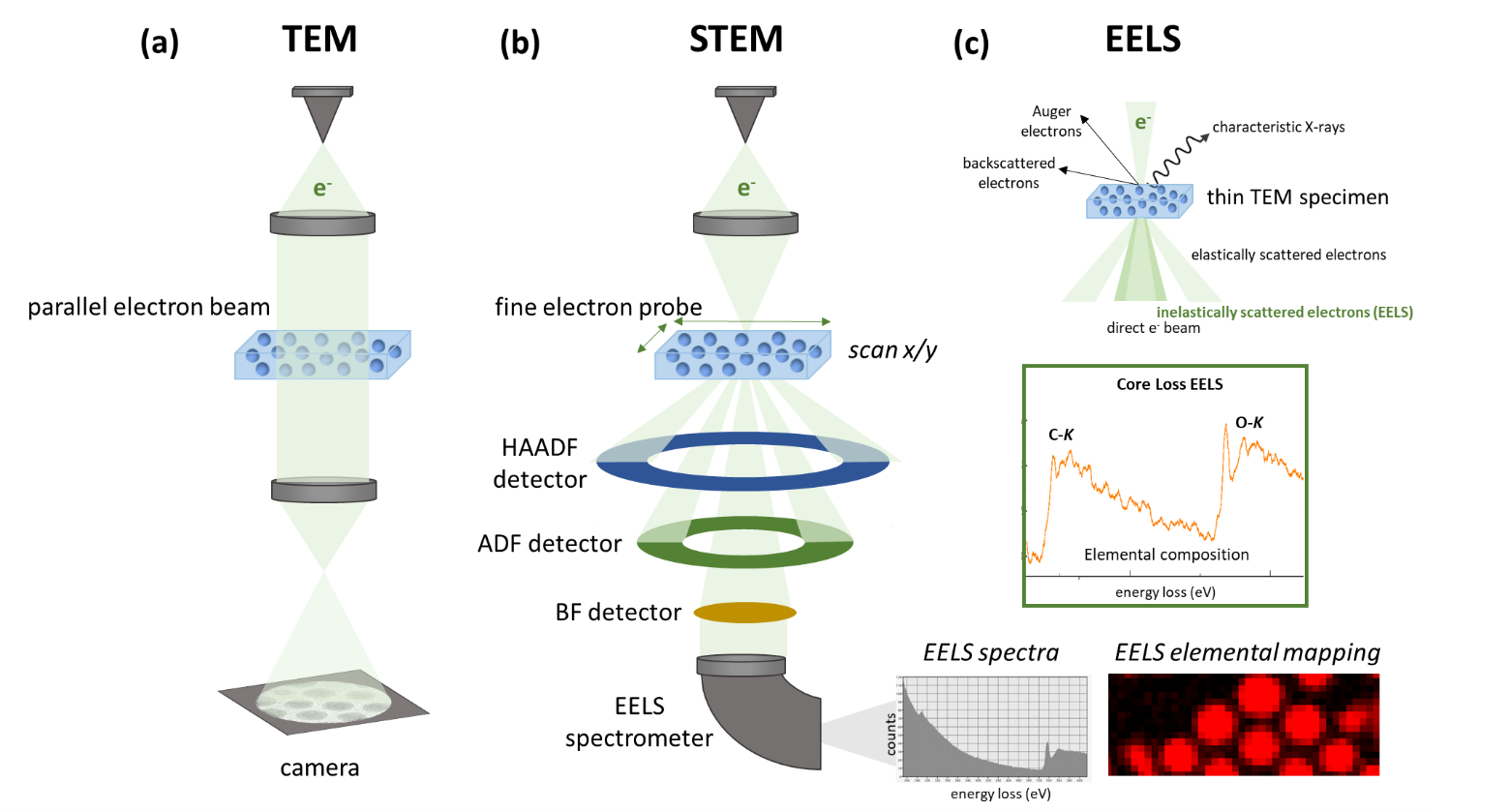


Figure S1 Comparison of beam propagation in (a) TEM (parallel electron beam) and (b) STEM (fine electron probe) modes, with the STEM also coupled to (c) an EELS spectrometer. EELS signal can be collected simultaneously and sequentially to provide high spatial chemical and structural information alongside the image.


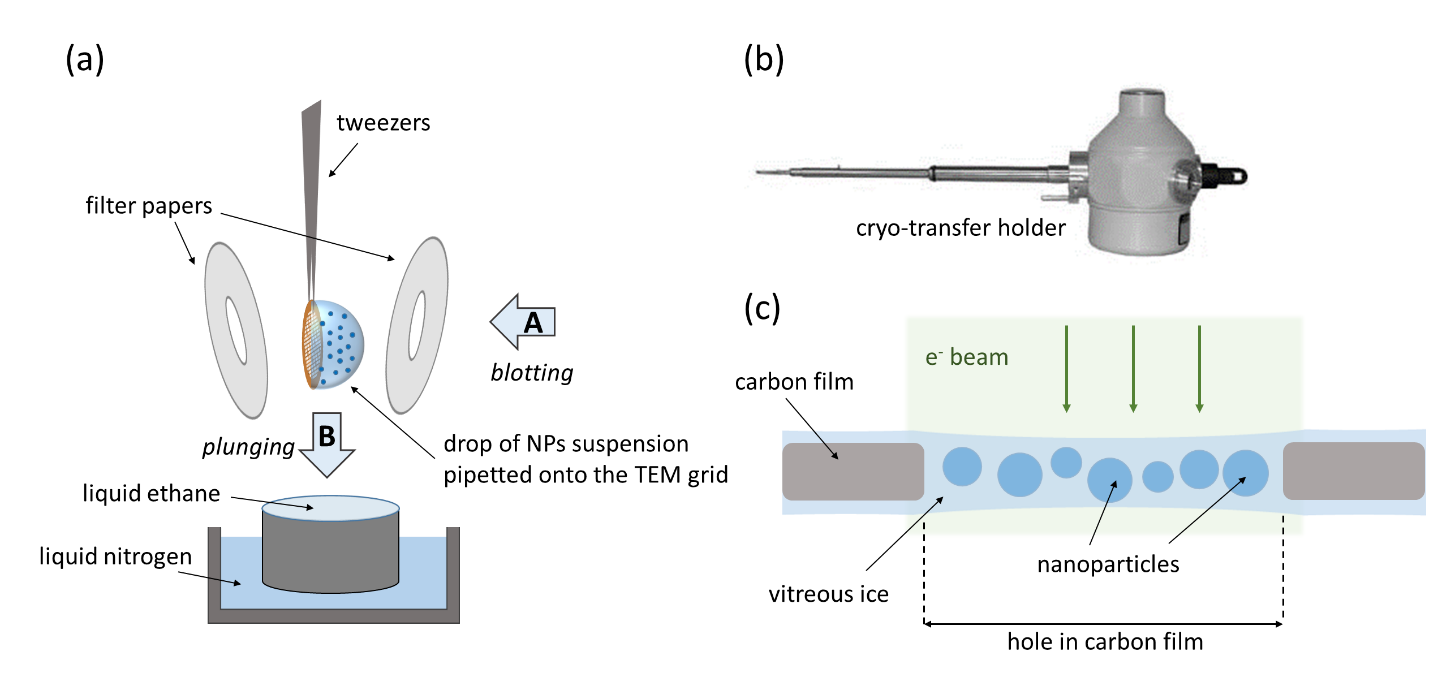


Figure S2 Schematic representation of cryo-EM sample preparation: (a) the drop of suspension is loaded onto EM grid and blotted (A) and then the grid is rapidly plunge frozen into liquefied ethane (B) and (b) transferred into a cryo-transfer holder under liquid nitrogen. (c) Cryo-EM thin film of dispersion can be then analysed via cryo-(S)TEM techniques.

**References**

1. Egerton, R., Physical principles of electron microscopy: An introduction to TEM, SEM, and AEM. 2005.

2. Williams, D. and C. Carter, Transmission Electron Microscopy. 2009, New York: Plenum Press.

3. Brydson, R., Electron Energy Loss Spectroscopy (1st ed.). 2001: Garland Science.

4. Egerton, R., Electron Energy-Loss Spectroscopy in the Electron Microscope, 3rd Edition (Springer, 2011). 2011.

5. Shen, P.S., The 2017 Nobel Prize in Chemistry: cryo-EM comes of age. Analytical and Bioanalytical Chemistry, 2018. 410(8): p. 2053-2057.

6. De Yoreo, J.J. and S. N. A. J. M, Investigating materials formation with liquid-phase and cryogenic TEM. Nature Reviews Materials, 2016. 1(8): p. 16035.

**Supporting Information 2**

**Table S1 BF-TEM imaging experimental condition.**

| **Pixel size**  **(nm x nm)** | **Pixel image size**  **(px x px)** | **Cumulative electron fluence per image**  **(e^-^/Å^2^)** | **Corresponding Figures** |
| --- | --- | --- | --- |
| 1.5 x 1.5 | 4096 x 4096 | ~ 2 | Figure 7a  Figure 9a (right) |
| 0.85 x 0.85 | 4096 x 4096 | ~ 2 | Figure 2a |
| 0.2 x 0.2 | 4096 x 4096 | ~ 10 | Figure 2b and c  Figure 3a,b,c  Figure 5a  Figure 9a (left and mid) |

**Table S2 STEM-ADF and STEM-EELS experimental condition.**

|  | | **Pixel size**  **(nm x nm)** | **Probe current**  **(pA)** | **Dwell time** | **Cumulative electron fluence per scan**  **(e^-^/Å^2^)** | **Corresponding Figures** |
| --- | --- | --- | --- | --- | --- | --- |
| **ADF** | 1.32 x 1.32 | | 40 | 40 µs | ~ 57 | Figure 5b and c  5d and e (processed using a high pass filter in the Thermo Scientific Velox™) |
|  | 2.7 x 2.7 | | 33 | 20 µs | ~ 6 | Figure 6a (left) |
| **EELS** | 12 x 12 | | 33 | 0.05 s | ~ 715 | Figure 6a  Figure 7a and b  Figure 8  Figure 9b |

**Supporting information 3**

**Table S3 Measurement of the corresponding particles using in focus and 4 µm under-focus condition. A two-tail P-test was performed on both datasets.**

|  | **In focus** | **4 µm defocus** |
| --- | --- | --- |
| *Measurement of the corresponding particles* | 63.9 | 65.5 |
|  | 70.4 | 68.5 |
|  | 74.5 | 73.2 |
|  | 69.6 | 69.1 |
|  | 75.1 | 73.8 |
|  | 75.1 | 73.2 |
|  | 74 | 71.4 |
|  | 70.4 | 70.2 |
|  | 72.9 | 75 |
|  | 78.1 | 78.5 |
|  | 79.8 | 79.7 |
| *Mean* | **73.9** | **73.2** |
| *Variance* | 10.0 | 12.6 |
| *Observations* | 11 | 11 |
| *P(T<=t) two-tail* | **0.097** |  |
|  | > 0.05  **P(T>=t)** | *null hypothesis is not rejected* |

**Supporting Information 4**

**Figure S3 Measurement procedure to calculate radius of (a) layer 1, (b) layer 2 and (c) layer 3.**


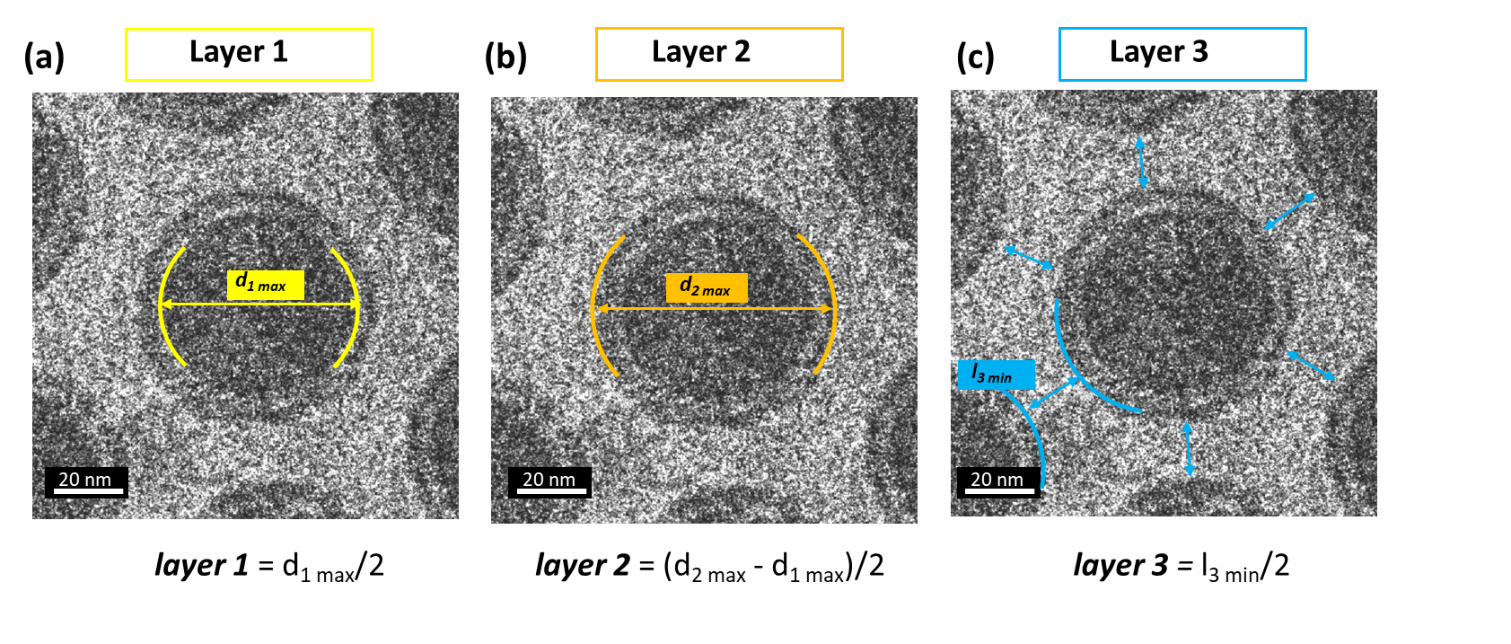


1. **Layer 1**

- the diameter is the maximum Feret diameter
- the radius is half the maximum Feret diameter

1. **Layer 2**

- the thickness (radius) is half of the difference between the two Feret diameters of layer 1 and 2.

1. **Layer 3**

- the thickness (radius) is half of the smallest distance between adjacent particles

**Supporting Information 5**

**Figure S4 Principal component analysis: (a) scree plot used for identification of (b)-(f) first five significant components that are presented as loading (spectra) and scores (image).**


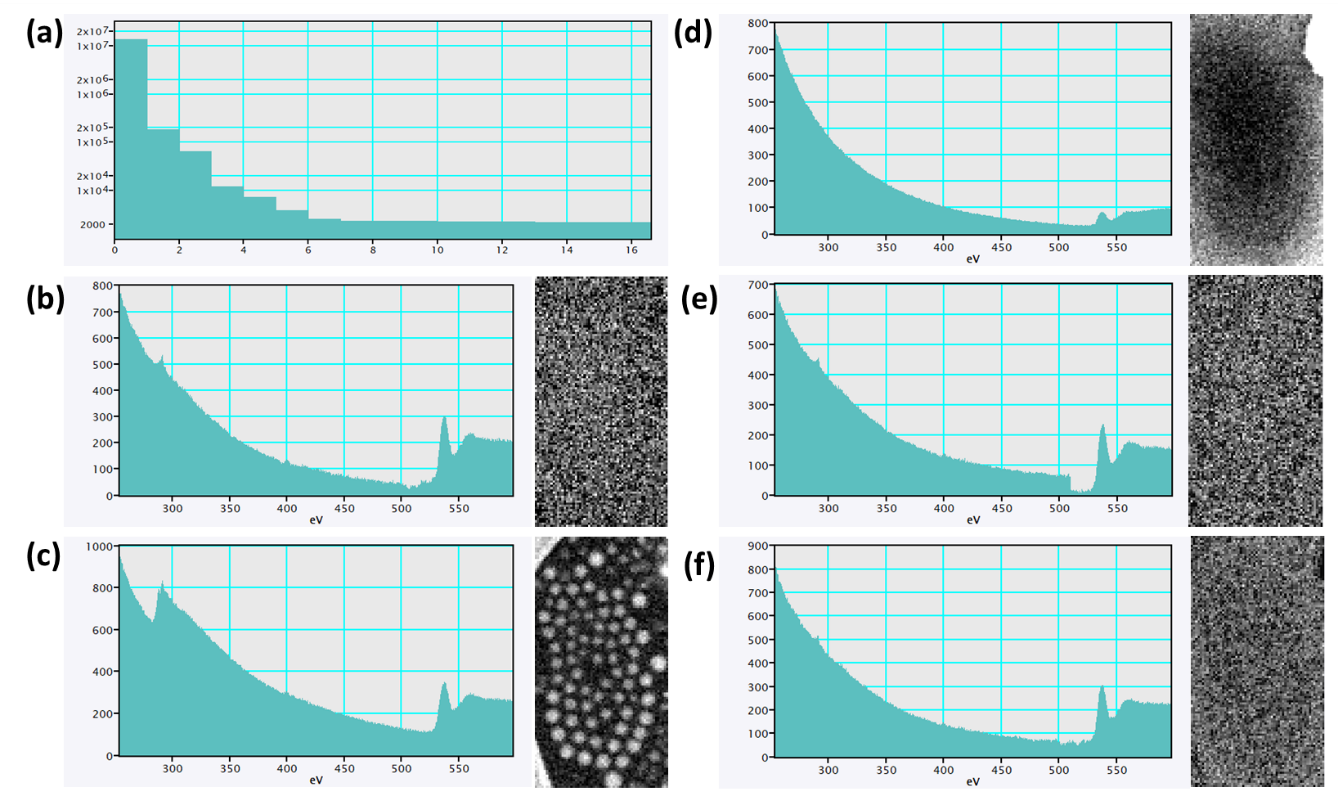


Identification of significant component:

- Scree plot (Fig S4a) suggests 3-5 significant components.
- The first component (Fig S4b) consists the mean STEM-EEL spectrum and C-*K*, N-*K* and O-*K* edges are distinguishable.
- Second component (Fig S4c) is clearly related to C-*K* and O-*K* edge, but N-*K* edge is also visible.
- The subsequent 3rd, 4th and 5th component (Fig S4d-f) can be attributed to mainly to O-*K* as oxygen dominates in cryogenic specimen, however these can also be related to the minor signal from C-*K* (all) and N-*K* (4^th^ and 5^th^ components, Figure S4e-f).
- However, the number of components is commonly lower than the expected factors due to the noise present in the data. In order to avoid artefacts related to overestimate the number of components, only first two meaningful components (Figure S4b-c) are used to provide denoised STEM-EELS mapping.
